# Case report: Superficial cervicovaginal myofibroblastoma of the cervix with endometrial carcinoma

**DOI:** 10.3389/fmed.2023.1160273

**Published:** 2023-04-04

**Authors:** Xiaowei Zhang, Bifei Huang, Junqiang Du

**Affiliations:** ^1^Department of Pathology, Affiliated Dongyang Hospital of Wenzhou Medical University, Dongyang, Zhejiang, China; ^2^Department of Gynaecology, Affiliated Dongyang Hospital of Wenzhou Medical University, Dongyang, Zhejiang, China

**Keywords:** cervix, neoplasm, benign, endometrioid carcinoma, surgery

## Abstract

**Introduction:**

A superficial cervical vaginal myofibroblastoma is a rare mesenchymal tumor, originating from the superficial stroma of the vagina and cervix. This study reports a patient, who was diagnosed with endometrioid carcinoma and a concomitant benign superficial cervicovaginal myofibroblastoma.

**Case presentation:**

A 53-year-old female with endometrial carcinoma was admitted to the Department of Gynecology of our hospital. She had a history of breast cancer on May 23, 2010, and took toremifene citrate for 41 months. Radical resection of the endometrial carcinoma was performed at our hospital. Based on the pathological findings, she was postoperatively diagnosed with endometrial adenocarcinoma with superficial cervical vaginal myofibroblastoma. The patient continued receiving postoperative breast cancer treatment. She underwent follow-up for 23 months. No recurrence or metastasis of the endometrial cancer or superficial cervical vaginal myofibroblastoma was observed.

**Conclusion:**

There were similarities between superficial cervical vaginal myofibroblastoma and other mesenchymal tumors of the female genital tract. Superficial cervical vaginal myofibroblastomas have a good prognosis, and the combination of tissue morphology and immunohistochemistry helped establish a definitive diagnosis.

## 1. Introduction

A superficial cervical vaginal myofibroblastoma (SCVM) is a mesenchymal tumor, originating from the superficial stroma of the vaginal and cervical mucosa. Clinically, SCVM has been confused with other soft tissue tumors of the female genital tract. This study reports the first case of endometrioid carcinoma, associated with SCVM. The findings of this study will contribute to further understanding of this rare disease.

## 2. Case description

### 2.1. Patient history

A 53-year-old woman was admitted to the hospital on June 10, 2019, 20 days after being diagnosed with uterine endometrioid carcinoma. The patient went into menopause at the age of 49 years with no abnormal postmenopausal vaginal bleeding. She previously underwent breast-conserving surgery for infiltrating ductal carcinoma of the left breast with left axillary lymph node metastasis at our hospital on May 23, 2010. The pathological examination revealed infiltrating ductal carcinoma (grade 1) of the left breast with axillary lymph node metastasis. Postoperatively, she received chemotherapy, radiotherapy, and endocrine therapy, consisting of a non-steroidal antiestrogen, toremifene citrate, for 41 months.

### 2.2. Gynecological examination

The cervix was glossy, with no noted erosion or tenderness. A pedunculated mass, measuring 2.0 × 2.0 cm, was palpated in the smooth non-tender cervix.

### 2.3. Investigations

The initial vaginal ultrasound examination, performed in our hospital, showed an anteverted uterus, measuring 117 × 123 × 68 mm, with a dark liquid area in the uterine cavity, which measured 110 × 104 × 54 mm. Several hyperechoic clusters were also appreciated, and the largest lesion measured 67 × 41 mm. Color Doppler flow imaging showed slightly rich blood flow signals in the uterine cavity. Curettage was performed, and the pathological examination revealed G1 endometrioid adenocarcinoma.

**Figure 1:**
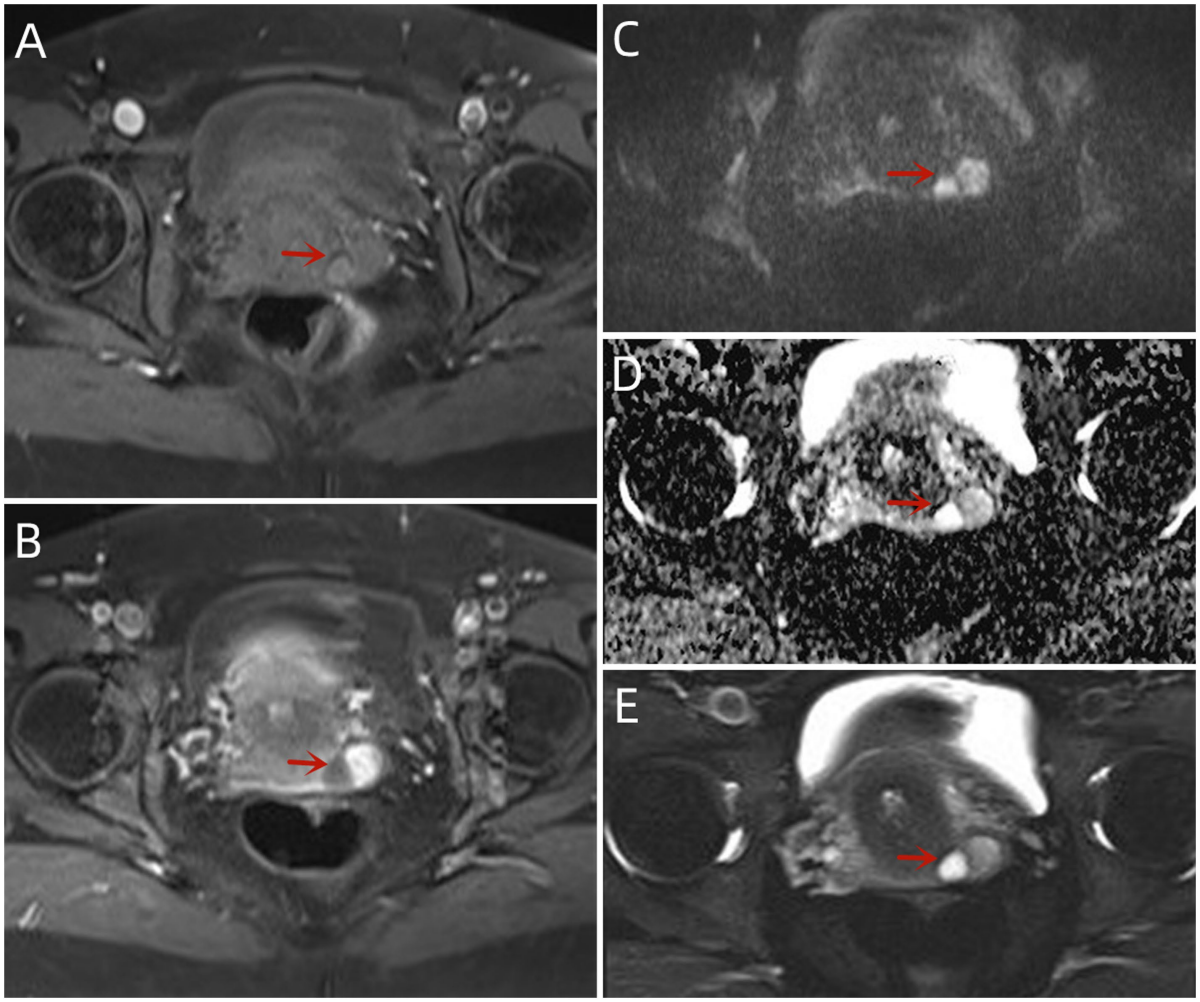
MRI findings of this case: **(A)** The lesions with the T2-FS sequence exhibited an uneven hypersignal. The right side exhibited a T2 hypersignal, similar to fluid. Magnetic resonance imaging findings of this case: **(B,C)** Both diffusion-weighted imaging (B800) and apparent diffusion coefficient lesions exhibited high signals with an unrestricted diffusion. **(D)** The T1-FS lesions had uneven and slightly hyperactive signals. **(E)** Enhancement of the left side of the lesion was observed after intravenous injection of contrast medium.

Magnetic resonance imaging showed a significantly enlarged uterus. Multiple liquid signal shadows were observed in the uterine cavity. It exhibited a slightly high signal on T1-weighted imaging (WI) and a high signal on T2WI, and the junction zone was intact. Multiple nodular abnormal signal foci were observed in the uterine cavity with a slightly low signal on T1WI and a slightly high signal on T2WI. The larger signal size measured 3.7 × 1.7 cm. Diffusely arranged lesions were observed at the uterine base, and no contrast enhancement was detected. The remaining lesions had a limited diffusion, and significant enhancement was observed after contrast injection. Small, round, long T1 and T2 signal foci without apparent enhancement were observed in the cervix (Figure 1).

### 2.4. Treatment and outcome

**Figure 2:**
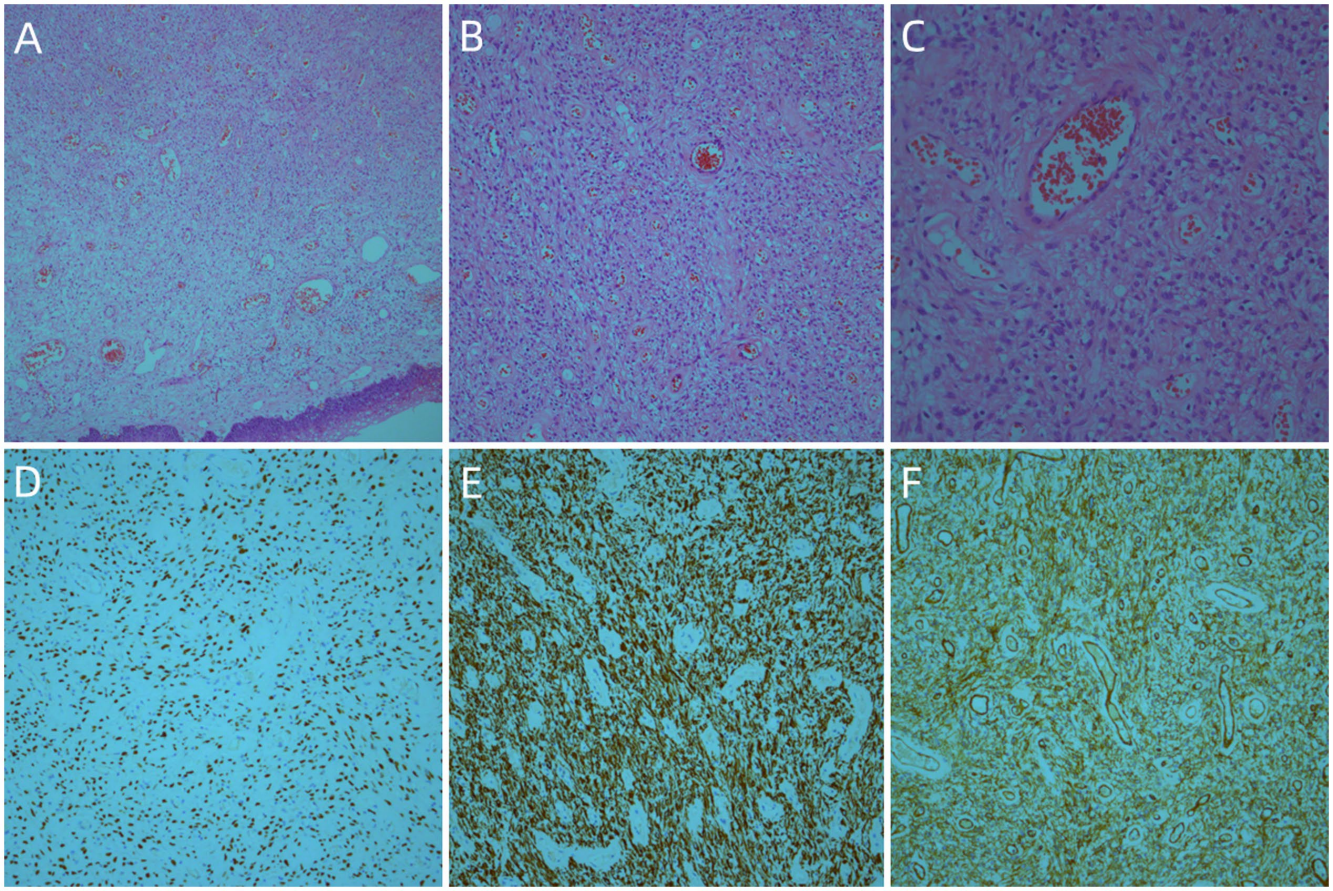
Histologic findings of this case: The tumor was located in the submucosa, and the tumor cells in the superficial area were relatively sparse. The tumor cells consisted of spindle cells and stellate cells with similar morphologies (**A**, ×50) (**B**, ×100) (**C**, ×200). Immunohistochemistry (IHC) staining revealed a strong expression for estrogen receptors (**D**, ×100), desmin (**E**, ×100), and CD34 (**F**, ×100).

Radical surgery for endometrial carcinoma was performed on June 12, 2019. The postoperative pathological examination revealed G1 endometrioid adenocarcinoma, located in the inner mucosal layer. Metastatic lesions were not detected among the 15 harvested lymph nodes. Additionally, intrauterine leiomyomata were observed, and the cervical histology was consistent with SCVM. On immunohistochemical staining, the tumor cells of the SCVM expressed estrogen receptors, progesterone receptors, vimentin, desmin, and CD34. However, they did not express S-100 and actin. The tumor cells had a Ki-67 index of 1% (Figure 2).

Postoperatively, the patient continued receiving hormonal therapy and chemotherapy for breast cancer while under monitoring for 23 months. Neither recurrence nor distant metastasis of the endometrial cancer or SCVM was detected.

## 3. Discussion

Superficial cervical vaginal myofibroblastoma is a benign mesenchymal tumor of the vagina and cervix. Soft tissue tumors of the female genital tract, such as smooth muscle tumors, rhabdomyoma, angiomyofibroblastoma, and invasive hemangiomasculoma, need to be clinically identified. Laskin et al. reported 14 cases of subepithelial mesenchymal tumors in the female genital tract (1). Superficial cervical vaginal myofibroblastoma is rare with only 57 reported cases, involving the female genital tract (2). This was the first report of concomitant SCVM and endometrioid carcinoma in a patient with a history of breast carcinoma. The pathogenesis of SCVM remains unclear. Some patients had a history of tamoxifen or hormone replacement therapy use (3). However, the role of hormonal therapy in the tumorigenesis and development of this disease requires further investigation. According to a previous hypothesis, the subepithelial mesenchymal cells in the lower female reproductive tract were positive for estrogen and progesterone. These mesenchymal cells served as the origin for mesenchymal tumors (4). Some studies suggested that SCVM was closely related to aggressive angiomyxoma, superficial angiomyxoma, cellular angiofibroma, angiomyofibroblastoma, and fibroepithelial polyps, lesions that were also commonly found in the female genital tract. They possibly shared a common tissue origin, particularly specialized mesenchymal pluripotent stem cells in the perivascular area of the female genital tract (5–8).

Superficial cervical vaginal myofibroblastoma of the female genital tract occurs mainly in the vagina, sometimes in the cervix, and occasionally in the vulva. The age of onset ranges from 23 to 80 years, with an average of 55 years. Most patients present with isolated masses, while few present with multiple nodules. Patients with this disease are typically asymptomatic. Few patients present with vaginal fluid discharge or bleeding, and the mass can be extruded from the vagina. In the present case, the patient was diagnosed with an endometrioid carcinoma and a concomitant SCVM. Since she also had breast cancer, she was taking an endocrine drug, toremifene citrate, which likely contributed to the pathogenesis of her endometrial endometrioid carcinoma and SCVM.

The imaging examination yielded non-specific findings. The ultrasound examination showed hyperechoic solid nodules with abundant blood signals. On magnetic resonance imaging, the lesion exhibited a muscle isosignal on T1WI, medium signal with high edge signal on T2WI, and high signal on Short TI Inversion Recovery sequence (9). The diagnosis of SCVM was based on the pathological findings. It presented as a polypoid mass with or without envelopment. The tumor size ranged from 1.0 to 6.5 cm, with an average diameter of 2.7 cm and a median diameter of 2.0 cm. The section surface was shiny or viscous and gray or pink in color. It had a soft-to-medium texture. Microscopically, the tumor was located in the submucosa and had a well-defined circular or oval shape. It was incompletely encapsulated. The surface squamous epithelium may be lifted by the tumor, forming a semi-circular or polyp shape. In most cases, the tumor cells in the superficial area were sparse with mucoid or edematous interstitium. In contrast, the tumor cells in the central area were dense, with more collagen fibers in the interstitium. Immunohistochemistry showed vimentin and desmin expression among tumor cells, while some expressed CD34. Estrogen and progesterone receptors were also expressed by tumors, suggesting a tumor–hormone association. The α-smooth muscle actin and MSA were partially expressed, but cytokeratin, epithelial membrane antigen, S-100, Calponin, CD99, and B-cell lymphoma-2 were not (10, 11). The primary differential diagnosis was invasive angiomyxoma, which is more common in young and middle-aged patients. The deep pelvic cavity and perineum exhibited invasive growth, with unclear tumor boundaries, mucoid tumor background, few cell components, blood vessels in the tumor, and spindle tumor cells around the blood vessels. Immunohistochemistry helped distinguish SCVM from invasive angiomyxoma.

Superficial cervical vaginal myofibroblastoma is a benign mesenchymal tumor with a good prognosis. Complete resection of the mass is the preferred management, and patients improve after complete local resection. Most patients do not experience recurrence or distant metastasis after resection. Improper excision is the most common cause of recurrence. Stewart et al. (12) reported a case of local recurrence in a patient with SCVM, occurring 9 years after a complete initial surgical resection. The patient in the present case was diagnosed with endometrioid cancer, based on the curettage findings. Thus, radical resection of the endometrial carcinoma was performed. Since the patient also had breast carcinoma, she continued receiving breast carcinoma treatment after radical endometrial carcinoma surgery. After 23 months of postoperative follow-up, neither tumor recurrence nor distant metastasis was detected.

## 4. Conclusion

This study presented a case of endometrioid carcinoma with SCVM. Both early-stage endometrioid carcinoma and SCVM, a rare mesenchymal tumor, have a favorable prognosis. Complete resection is recommended for patients with SCVM to ensure sufficient resection margins. Close monitoring after the surgery is also recommended. The estrogen receptor modifier of toremifen citrate promotes the tumorigenesis and development of endometrial cancer. However, SCVM is hormone-dependent, and develops due to the neoplastic proliferation of hormone-responsive stromal cells. The role of estrogen receptor modifiers in the development of endometrial cancer and SCVM needs further investigation.

## Data availability statement

The original contributions presented in the study are included in the article/supplementary material, further inquiries can be directed to the corresponding authors.

## Ethics statement

Ethical review and approval was not required for the study on human participants in accordance with the local legislation and institutional requirements. Written informed consent from the patients was not required to participate in this study in accordance with the national legislation and the institutional requirements. Written informed consent was obtained from the patient for the publication of any potentially identifiable images or data included in this article.

## Author contributions

XZ and JD acquired the data. BH analyzed the pathological data. JD analyzed the imaging data. XZ, JD, and BH prepared the manuscript. All authors contributed to the article and approved the submitted version.

## Conflict of interest

The authors declare that the research was conducted in the absence of any commercial or financial relationships that could be construed as a potential conflict of interest.

## Publisher’s note

All claims expressed in this article are solely those of the authors and do not necessarily represent those of their affiliated organizations, or those of the publisher, the editors and the reviewers. Any product that may be evaluated in this article, or claim that may be made by its manufacturer, is not guaranteed or endorsed by the publisher.

## Abbreviations

SCVM: superficial cervicovaginal myofibroblastoma
MRI: magnetic resonance imaging
T1WI: T1-weighted imaging
T2WI: T2-weighted imaging
ER: estrogen receptors
PR: progesterone-receptors
IHC: immunohistochemistry
DWI: diffusion weighted imaging
ADC: apparent diffusion coefficient
T1-FS: T1-fat saturation
T2-FS: T2-fat saturation

## References

[1] Laskin WB Fetsch JF Tavassoli FA. Superficial cervicovaginal myofibroblastoma: fourteen cases of a distinctive mesenchymal tumor arising from the specialized subepithelial stroma of the lower female genital tract. Hum Pathol. (2001) 32:715–25. doi: 10.1053/hupa.2001.25588, PMID: 11486170

[2] Wang Y Sun M Wang J. Superficial vaginal myofibroblastoma with mushroom-like appearance: a case report with colposcopic findings and literature review. Front Oncol. (2022) 12:1024173. doi: 10.3389/fonc.2022.1024173, PMID: 36387153 PMC9647032

[3] Ganesan R McCluggage WG Hirschowitz L Rollason TP. Superficial myofibroblastoma of the lower female genital tract: report of a series including tumours with a vulval location. Histopathology. (2005) 46:137–43. doi: 10.1111/j.1365-2559.2005.02063.x, PMID: 15693885

[4] Zhang M Shi P Zhou B Liu J Li L. Superficial myofibroblastoma of the lower female genital tract: a clinicopathological analysis of 15 cases. Ann Diagn Pathol. (2022) 60:152010. doi: 10.1016/j.anndiagpath.2022.152010, PMID: 35907316

[5] Olinici CD Crişan D Zolog A Puşcaş M. Vaginal superficial myofibroblastoma. Case report and review of the literature. Romanian J Morphol Embryol. (2007) 48:165–70. 17641804

[6] Nucci MR. Mesenchymal lesions of the lower Ge nital tract. Surg Pathol Clin. (2009) 2:603–23. doi: 10.1016/j.path.2009.08.014, PMID: 26838773

[7] Tajiri R Shiba E Iwamura R Kubo C Nawata A Harada H. Potential pathogenetic link between angiomyofibroblastoma and superficial myofibroblastoma in the female lower genital tract based on a novel MTG1-CYP2E1 fusion. Mod Pathol. (2021) 34:2222–8. doi: 10.1038/s41379-021-00886-8, PMID: 34385605

[8] Schoolmeester JK Fritchie KJ. Genital soft tissue tumors. J Cutan Pathol. (2015) 42:441–51. doi: 10.1111/cup.12507 25925211

[9] Atinga A El-Bahrawy M Stewart V Bharwani N. Superficial myofibroblastoma of the genital tract: a case report of the imaging findings. BJR Case Rep. (2019) 5:20180057. doi: 10.1259/bjrcr.20180057, PMID: 31131129 PMC6519501

[10] McCluggage WG. Recent developments in vulvovaginal pathology. Histopathology. (2009) 54:156–73. doi: 10.1111/j.1365-2559.2008.03098.x, PMID: 18637148

[11] Magro G Caltabiano R Kacerovská D Vecchio GM Kazakov D Michal M. Vulvovaginal myofibroblastoma: expanding the morphological and immunohistochemical spectrum. A clinicopathologic study of 10 cases. Hum Pathol. (2012) 43:243–53. doi: 10.1016/j.humpath.2011.04.027, PMID: 21820148

[12] Stewart CJ Amanuel B Brennan BA Jain S Rajakaruna R Wallace S. Superficial cervico-vaginal myofibroblastoma: a report of five cases. Pathology. (2005) 37:144–8. doi: 10.1080/00313020500058284, PMID: 16028842

